# A quick, cheap, and reliable protocol for immunofluorescence of pluripotent and differentiating mouse embryonic stem cells in 2D and 3D colonies

**DOI:** 10.1016/j.xpro.2022.102000

**Published:** 2023-01-20

**Authors:** Snježana Kodba, Agathe Chaigne

**Affiliations:** 1 Department of Cell Biology, Neurobiology and Biophysics, Utrecht University, 3584 CH Utrecht, the Netherlands

**Keywords:** Cell Biology, Developmental biology, Stem Cells

## Abstract

Immunofluorescent labeling is a widely used method to visualize endogenous proteins. It can be expensive and difficult to stain mouse embryonic stem cells (mESCs) because they require expensive growth media, prefer specific substrates, grow in 3D, and have loose cell-substrate adhesion. Here we propose a half-a-day, cheap, easy-to-follow, and reproducible protocol for immunofluorescence of mESCs. This protocol has been streamlined to allow a fast visualization of the investigated proteins, and we provide tips specific to stem cell culture.

For complete details on the use and execution of this protocol, please refer to Chaigne et al. (2021).^1^

## Highlights

• Quick and reliable protocol for immunofluorescence of stem cells
• Tips for stem cell culture in 2D and 3D colonies
• Cheap immunostaining with reduced antibody usage
• Adjustments of the standard protocol for better imaging of cell cortex proteins

## Abstract (editor highlights)

Publisher’s note: Undertaking any experimental protocol requires adherence to local institutional guidelines for laboratory safety and ethics.

## Graphical abstract

**Figure:**
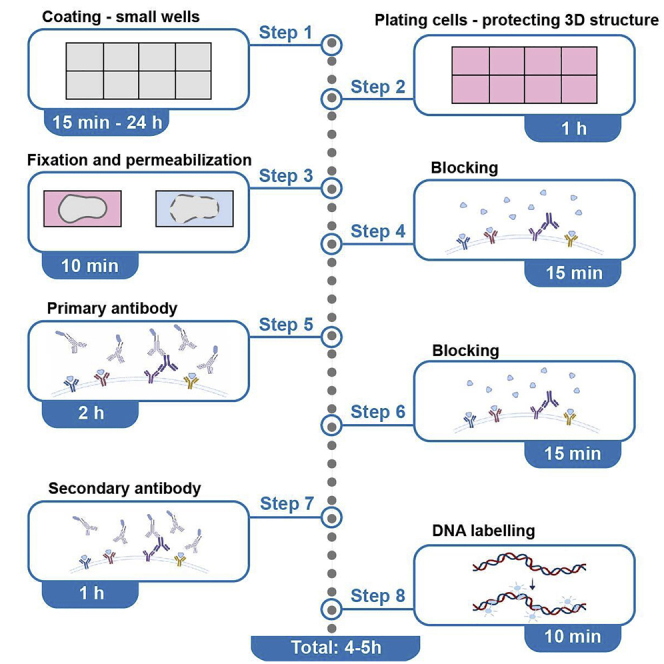

## Before you begin

The goal of this protocol is to stain endogenous proteins with a cheap and fast method while preserving the 3D structure of colonies of cells. This protocol is also suitable for 3D organoid cultures embedded in Matrigel. The main advantage of this method is that is uses very small wells allowing the use of small amounts of media and antibodies which reduces the cost, and preserves the 3D architecture.

The immunofluorescence protocol below describes the specific steps using mouse embryonic stem cells (mESC). However, this protocol can also be used for other mammalian cell cultures. Before you start, you need to prepare the specific cell media required for the experiment. Naïve mouse embryonic stem cells are grown in 2i/LIF conditions (containing two inhibitors of the Erk1/2 signaling pathway and of Glycogen Synthase Kinase 3 (GSK3) and Leukemia Inhibitory Factor (LIF). Cells exiting naïve pluripotency are cultured in N2B27. The culture media conditions for mouse embryonic stem cells have been developed in the Smith lab.^2^

Mouse embryonic stem cells can be plated on two different substrates. If you choose to plate them on gelatin, follow option 1. The main advantage of plating cells on gelatin is that it maintains the 3D organization of mESC. It does, however, make them harder to image. On the other hand, option 2 offers plating cells on laminin where they grow in 2D, allowing faster imaging and easier visualization of proteins. Here we present a protocol for cultures that have been growing during 48 h, but numbers can in principle be scaled linearly for shorter culture (24 h, 36 h) or longer culture (72 h, 96 h). Culture longer than 96 h is not recommended.

We will discuss two variations of the protocol: the first one is a standard protocol which works for most of the proteins (here we propose to stain for α-Tubulin and actin using Phalloidin), and the second is a variant of that protocol which is specified for cortical proteins like NuMA (here we propose to stain for α-Tubulin and NuMA).

### Culture of 3D colony of mouse embryonic stem cells

**Timing: 1 h**

1. Prepare fresh coating agent and media for cells (once prepared, gelatin can be used for 6 months, laminin has to be used immediately, 2i/LIF can be used for 2 weeks and N2B27 can be used for 3 weeks).
2. To prepare 0.1% gelatin use a new bottle of PBS.
  a. Weigh 0.5 *g* gelatin in a 50 mL tube and add 50 mL PBS.
  b. Shake well.
  c. Dissolve for 30 min in a 37°C water bath.
  d. Filter using a 50 mL syringe and a 0.2 μm filter in a 50 mL tube.
  e. Add back to the PBS bottle.
3. For naïve mouse embryonic stem cells, use 2i/LIF media.
4. Pre-warm media and Accutase™.
5. Coat the glass-bottom 8-well IBIDI plate wells with 130 μL of 0.1% gelatin 15 min before seeding cells.

***Note:*** The wells need to be covered with gelatin for at least 15 min at room temperature (20°C–25°C) prior to seeding the cells. These plates have small wells which enable less antibody usage per sample. Bigger plates, e.g., 24-well or 96-well plate can also be used for higher throughput.

Troubleshooting 1. (see below).

### Alternative: Culture of 2D colony of mouse embryonic stem cells

**Timing: 1 h not including overnight coating**

***Alternative:*** Prepare the laminin solution by mixing 20 μL Laminin in 980 μL DPBS. Prepare the glass-bottom 8-well IBIDI laminin plate by adding the 130 μL of the laminin solution. Incubate at 37°C overnight (16 h) or for a minimum of 4 h.

### Plating of the cells

The following steps are the same for both options.

6. Remove media from the cells and add prewarmed Accutase™.
7. Leave it on the cells for 3 min in the incubator at 37°C and at 7% CO_2_. Accutase^TM^ can be left a few more min on the cells if need be.

***Note:*** 1 ml of Accutase™ is enough for a 10 cm^2^ cell culture dish. Accutase™ needs to be stored at −20°C long term or 4°C short term. Keeping it at room temperature for longer time will inactivate the enzyme.

8. Carefully resuspend the cells and pipette up and down to obtain a single cell solution.

***Note:*** Check under the microscope if the cells are not in clumps before proceeding to the next step. If cells are still in clumps, leave the Accutase™ for a few extra min, not exceeding 10.

9. Add the cells to a tube containing 5 mL DMEM F12 + 5% BSA.
10. Centrifuge the cells for 3 min at 1000 rpm/1500 *g*.
11. Remove the media containing Accutase™ and leave the pellet of cells.
12. Add 1 mL of DMEM F12 + 5% BSA and resuspend the cells.
13. Remove the coating from the wells which you have previously coated.
14. Add enough media (∼200 μL per well) to cover the bottom of the wells.
15. Plate the cells at the required concentration in the wells.

***Note:*** When using 8-well plates with area of 2.20 cm^2^, plate 30000 cells for a 48 h culture.

16. After 48 h cells are ready to be stained.

***Note:*** Cells need to be attached to the bottom of the wells before you proceed with the protocol.

17. Before starting the staining, prepare all the solutions required. That includes fixative-permeabilization and blocking buffer. Details of the solutions can be found in the materials and equipment section.

## Key resources table

**Table:** 

REAGENT or RESOURCE	SOURCE	IDENTIFIER
**Antibodies**		

Rat monoclonal alpha tubulin (YL1/2)	Thermo Fisher Scientific	Cat# MA1-80017
Rabbit polyclonal NuMa	Abcam	Cat# ab36999
AlexaFluor 488 donkey anti-rabbit	Thermo Fisher Scientific	Cat# A32790
AlexaFluor 647 donkey anti-rat	Thermo Fisher Scientific	Cat# A48272

**Chemicals, peptides, and recombinant proteins**		

Gelatin from porcine skin	Sigma-Aldrich	Cat# G2500-500G
Laminin	Merck	Cat# 11243217001
Accutase™	Sigma-Aldrich	Cat# A6964
16% formaldehyde solution	Thermo Fisher Scientific	Cat# 28908
Dulbecco’s phosphate buffered saline (DPBS)	Sigma-Aldrich	Cat# D8537-500ML
Bovine serum albumin (BSA)	Capricorn Scientific	Cat# BSA-DG-500G
Trichloroacetic acid (TCA)	Merck	Cat# 8223421000
Hoechst 33342	Thermo Fisher Scientific	Cat# 34580
Skim milk powder for microbiology	Merck	Cat# 115363
Triton X-100	Sigma-Aldrich	Cat# 93443
PIPES	Sigma-Aldrich	Cat# P1851
Alexa Fluor™ 568 Phalloidin	Thermo Fisher Scientific	Cat# A12380
HEPES	Sigma-Aldrich	Cat# H3784
EGTA	Sigma-Aldrich	Cat #324626
MgCl2	Sigma-Aldrich	Cat #M8266
DMEM F12 media	Thermo Fisher Scientific	Cat# 11320033
Neurobasal media	Thermo Fisher Scientific	Cat# 21103049
L-glutamine	Thermo Fisher Scientific	Cat# 25030081
Penicillin/streptomycin	Thermo Fisher Scientific	Cat# 15140122
Gibco™ B-27™ Supplement (50X), minus vitamin A	Thermo Fisher Scientific	Cat# 12587010
Insulin, human recombinant, zinc solution	Thermo Fisher Scientific	Cat# 12585014
CHIRON	StemCell Technologies	Cat# 72054
PD 0325901	Sigma-Aldrich	Cat# PZ0162
ESGRO® Recombinant Mouse LIF Protein	Sigma-Aldrich	Cat# ESG1107
Βmercaptoethanol	Sigma-Aldrich	Cat# M6250-250ML
Apotransferrin	Sigma-Aldrich	Cat# T1147-500MG
DMEM Hams F12	Thermo Fisher Scientific	Cat# 11765054
Sodium selenate solution	Sigma-Aldrich	Cat# S5261
Putrescine solution	Sigma-Aldrich	Cat# P5780
Progesterone solution	Sigma-Aldrich	Cat# P0130

**Experimental models: Cell lines**		

Mouse embryonic stem cells: E14	Geijsen lab (LUMC Leiden)	N/A

**Other**		

μ-Slide 8 Well Glass Bottom	Ibidi	Cat# 80827

## Materials and equipment

### Preparation of N2

**Table:** 

Reagent	Final concentration	Amount
Apotransferrin	8.791 mg/mL	1 *g*
Water	N/A	10 mL
DMEM Hams F12	N/A	92.5 mL
BSA	0.66%	10 mL
Sodium selinate solution	3 μM	120 μL
Putrescine solution	1.688 mg/mL	1200 μL
Progesterone solution	2.08 μg/mL	390 μL
**Total**	**N/A**	**104.2 mL**

***Note:*** N2 can be stored for 2 years at −80°C.

### Components of 2i/LIF media

**Table:** 

Reagent	Final concentration	Amount
DMEM F12 media	N/A	25 mL
Neurobasal media	N/A	25 mL
BSA	1.2%	600 μL
L-glutamine	2 mM	550 μL
Penicillin/Streptomycin	1:100	500 μL
B27	1:100	500 μL
N2	1:200	250 μL
Insulin zinc	12.5 μg/ml	156.25 μL
CHIRON	3 μM	15 μL
PD 0325901	1 μM	5 μL
LIF	1000 U/mL	5 μL
βmercaptoethanol	0.1 mM	5.5 μL
**Total**	**N/A**	**52.9 mL**

***Note:*** 2i/LIF can be stored for 2 weeks at 4°C.

### Components of N2B27 media

**Table:** 

Reagent	Final concentration	Amount
DMEM F12 media	N/A	25 mL
Neurobasal media	N/A	25 mL
L-glutamine	2 mM	550 μL
Penicillin/Streptomycin	1:100	500 μL
B27	1:100	500 μL
N2	1:200	250 μL
Insulin zinc	N/A	156.25 μL
βmercaptoethanol	0.1 mM	5.5 μL
**Total**	**N/A**	**52.8 mL**

***Note:*** N2B27 can be stored for 3 weeks at 4°C.

### Immunofluorescence solutions

Fixation/permeabilization (option 1)

**Table:** 

Reagent	Final concentration	Amount
16% formaldehyde	4%	1 mL
Triton^TM^X-100	0.1%	5 μL
DPBS	N/A	3 mL

This solution can be jept for a week at 4°C.

Fixation and Permeabilization (option 2).

• Fixation (make fresh)

**Table:** 

Reagent	Final concentration	Amount
Trichloroacetic Acid (ice cold)	10%	0.1 mL of 100%
DPBS	N/A	0.9 mL

• Permeabilization (make fresh)

**Table:** 

Reagent	Final concentration	Amount
Triton^TM^X-100	0.5%	25 μL
DPBS	N/A	4 mL

Blocking (2 options).

• Option 1 (make fresh)

**Table:** 

Reagent	Final concentration	Amount
BSA	3%	1.5
DPBS	N/A	50 mL

• Option 2 (make fresh)

**Table:** 

Reagent	Final concentration	Amount
Skim milk	5%	2.5 *g*
DPBS	N/A	50 mL

### PHEM buffer

**Table:** 

Reagent	Final concentration	Amount
PIPES	60 mM	4.54 *g*
HEPES	25 mM	1.5 *g*
EGTA	10 mM	950 mg
MgCl_2_	2 mM	100 mg
H_2_O	N/A	Up to 250 mL

***Note:*** Adjust pH at 6.9. This solution can be stored for 6 months at RT.

**CRITICAL:** Work with formaldehyde and TCA under the fume hood.

***Alternatives:*** Paraformaldehyde can also be used instead of formaldehyde.

## Step-by-step method details

### Fixation and permeabilization

**Timing: 10–30 min**

Ideally, fixation should immobilize targeted antigens, but not disturb the cell structure.^3^ Here, we propose 2 options. Our main protocol proposes to fix and permeabilize at the same time for gain of time. Our second option uses TCA and proposes to fix then permeabilize. Most antibodies work well with simultaneous formaldehyde fixation/permeabilization.

***Note:*** If the simultaneous protocol does not work well, try to separate fixation and permeabilization. If this does not work well, try with a different fixative (0.2% glutaraldehyde, ice cold TCA or ice cold methanol).

1. Delicately remove the media from the well using a pipette at the corner of the well.

***Note:*** Remove the media and at the same time add the fixation/permeabilization solution (130 μL per well).

2. Incubate the cells in the solution for 10 min at room temperature (20°C–25°C).
  ***Note:*** To preserve better osmolarity, fixative and permeabilization solution can be prepared in PHEM buffer. Prepare fresh fixation/permeabilization solution each time you are doing the experiment.
  ***Alternative:*** For the 2-steps protocol use 10% ice cold TCA as a fixative. Fix cells for 20 min at 4°C.
3. Wash the fixative by rinsing the cells with PBS one time.
  ***Alternative:*** For the 2-steps protocol rinse the cells with DPBS three times for 5 min. Permeabilize the cells by incubating them in 0.5% Triton^TM^-X for 5 min. Rinse with DPBS (optional).

**CRITICAL:** This step should be done as fast as possible to prevent tissue or cell deformations. However, all rinses should be done carefully so the cells are not washed away from the well.

**Pause Point:** If needed, it is possible to pause after washing the fixative for up to 10 days. In that case, always rinse the cells three times for 5 min with PBS, wrap with stretch film to seal and store at 4°C.

Troubleshooting 2. (see below).

### Blocking

**Timing: 15 min**

The blocking step helps minimizing any unspecific antibody binding within the cell.^4^

4. Incubate cells with 200 μL 5% milk (blocking solution) for 15 min at room temperature.
  ***Alternative:*** For NuMA visualization incubate cells with 200 μL 3% BSA (alternative blocking solution) for 15 min at room temperature (20°C–25°C).

### Primary antibody

**Timing: 2 h**

To visualize the proteins of interest, use a specific antibody. You can use multiple primary antibodies in the same experiment. If using multiple primary antibodies, make sure that they originate from different species to avoid cross-reactivity when using secondary antibodies to visualize the proteins.

5. Prepare the antibody solution by diluting the primary antibodies in the blocking solution.

***Note:*** As a starting point, we find that most of the antibodies give a good result at a 1:200 ratio (1 μL of antibody can be diluted in 200 μL of the blocking solution). However, you can follow the instructions given for a specific antibody.

6. Incubate 130 μL of primary antibody solution for 2 h at room temperature (20°C–25°C) on a shaker at 20 rpm.

***Note:*** No humid chamber is needed. If needed, the primary antibodies can be incubated overnight (16 h) at 4°C.

Troubleshooting 3. (see below).

### Blocking

**Timing: 15 min**

7. Wash the primary antibody by rinsing the cells 3x with 200 μL DPBS.
8. Incubate cells with 200 μL of the chosen blocking solution for 15 min at room temperature (20°C–25°C).

### Secondary antibody

**Timing: 1 h**

The secondary antibodies should be specific to the species of the primary antibodies and have conjugated fluorophores in the wavelengths of interest. You can mix the secondary antibodies.

9. Prepare antibody solution by diluting secondary antibody in blocking solution.

***Note:*** As a starting point, we find that most of the antibodies give a good result at a 1:500 ratio (1 μL of antibody can be diluted in 500 μL of the blocking solution). However, you can follow the instructions given for a specific antibody.

10. Incubate with 130 μL of the secondary antibody solution for 1 h at room temperature (20°C–25°C) on a shaker at 20 rpm.
  ***Alternative:*** Phalloidin can be used to label actin at a 1:200 ratio and can be mixed with secondary antibodies.

***Note:*** Depending on the microscope used for visualization, you can use multiple different secondary antibodies. This depends on the laser separation of your microscope. We recommend using a simple confocal microscope with a good spectral separation.

Troubleshooting 3.

### DNA labeling

**Timing: 10 min**

If needed, the DNA can be stained as the final step of this protocol using Hoechst (1:10000) or DAPI (0.1 μg/mL).

11. Wash the secondary antibody by rinsing the cells 3x with DPBS.
12. Prepare Hoechst solution by mixing 0.5 μL of Hoechst and 5 mL of DPBS (1:10000).
13. Incubate cells with prepared Hoechst solution for 10 min at room temperature (20°C–25°C).
14. Wash the Hoechst solution by rinsing the cells 3x with DPBS.

**Figure 1:**
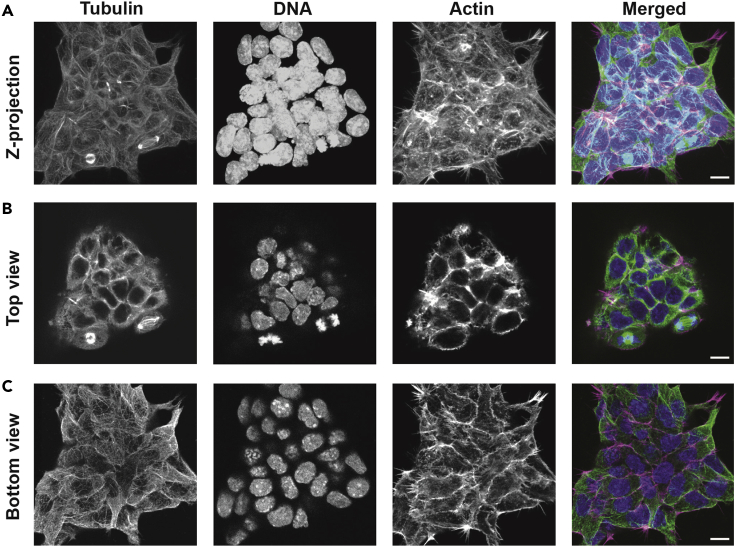
3D mouse embryonic stem cells colony cultured on gelatin during differentiation showing labeled tubulin, actin, and DNA (A) Z projection of the colony. (B) Top plane of the colony. (C) Bottom plane of the colony. The antibodies and dyes used for this staining are: α-Tubulin, Phalloidin 568 nm and Hoechst. Scale bar: 10 μm.

**Figure 2:**
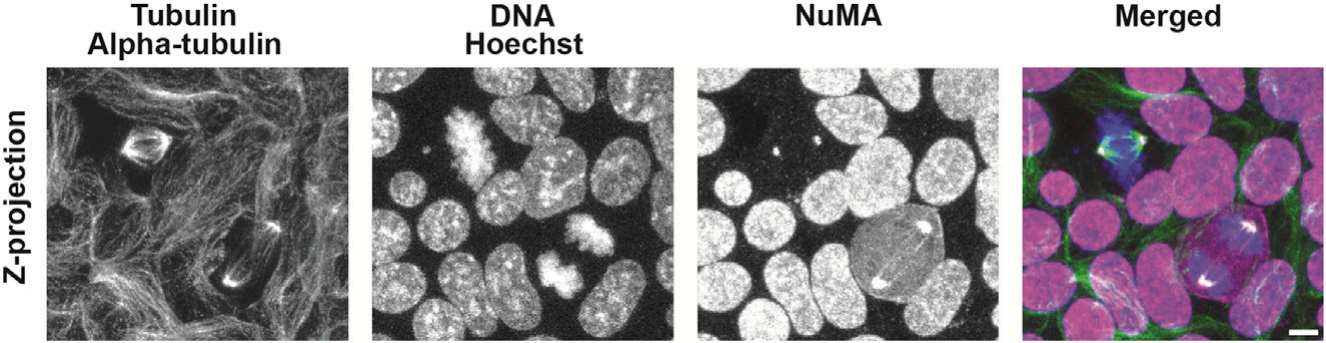
Pluripotent 2D mouse embryonic stem cells colony cultured on laminin showing labeled tubulin, NuMA, and DNA The antibodies and dyes used for this staining are: α-Tubulin, NuMA and Hoechst. Scale bar: 5 μm.

Leave the cells in DPBS 4°C. before imaging so the cells do not dry. For the best quality image cells within a few days (up to a week) of finishing this protocol. Examples can be found in Figure 1 (main protocol: 3D mouse embryonic stem cells colony cultured on gelatin during differentiation showing labeled tubulin, actin, and DNA) and Figure 2 (alternative protocol, Pluripotent 2D mouse embryonic stem cells colony cultured on laminin showing labeled tubulin, NuMA and DNA).

## Expected outcomes

Following this protocol should allow the quick and cheap visualization of a broad range of proteins. From top to finish the protocol can be reduced to ∼4 h. Using this protocol, mouse embryonic stem cells are cultured in small wells. This allows smaller media consumption, as well as using less antibodies for staining and allowing high-throughput testing of antibodies. Skipping the mounting step and imaging the cells directly in a well allow to preserve the 3D structure of colonies.

## Limitations

The usual limitations of immunofluorescence protocols apply. In particular, this protocol is only suitable for fixed cells, and some proteins are not well labeled by commercial antibodies. Autofluorescence, linked to the cell type, the fixation or blocking solutions, can interfere with fluorescence coming from the fluorochromes on secondary antibodies. A clear limitation of this specific protocol is the timing of imaging. Since cells are imaged directly in the culture well in PBS, the sample needs to be visualized as soon as possible to avoid possible bacterial contaminations.

## Troubleshooting

### Problem 1

When plating 3D culture of mouse embryonic stem cells, colonies seeded on gelatin can be easily washed from the well or coverslip during the washing steps.

### Potential solution

To improve adhesion, before coating the wells with gelatin, plasma activate the wells for 30s, using a plasma cleaner at maximal intensity. Plasma cleaning uses activated and ionized gas to break any impurities present on the substrate and allows for better adhesion of the coating reagent and the cells.

### Problem 2

Some molecules or proteins (e.g., Protein Regulator of Cytokinesis, PRC1) are not nicely visualized using 4% formaldehyde or 10% TCA fixation.

### Potential solution

Try different fixation protocols, e.g., ice cold methanol or 4% paraformaldehyde. The PHEM buffer increases the quality of staining of cytoskeletal protein. Ice cold methanol can be used to visualize cross-linking proteins such as PRC1, as formaldehyde and paraformaldehyde are cross-linking fixation agents which disrupt PRC1 structure. A quenching step can be added after fixation and permeabilization (15 min in 1 mg/mL^−1^ Sodium Borohydride in CMF-PBS (Ca and Mg free PBS)).

### Problem 3

Proteins or molecules of interest cannot be nicely visualized because.

• The background staining is too high;
• The intensity of the target protein is too low.

### Potential solution

• Increase the concentration of Triton-X^TM^ to increase the permeabilization.
• Change the blocking buffer (BSA or milk) and the antibody buffer. Increase the concentration of BSA or milk.
• Increase the concentration of secondary and/or primary antibody.

## Resource availability

### Lead contact

Agathe Chaigne (a.e.d.chaigne@uu.nl).

### Materials availability

This study did not generate new unique reagents.

## Data Availability

This study did not generate/analyze datasets/code.
